# Genome and Transcriptome Sequencing of *Populus* × *sibirica* Identified Sex-Associated Allele-Specific Expression of the *CLC* Gene

**DOI:** 10.3389/fgene.2021.676935

**Published:** 2021-08-11

**Authors:** Elena N. Pushkova, George S. Krasnov, Valentina A. Lakunina, Roman O. Novakovskiy, Liubov V. Povkhova, Ekaterina M. Dvorianinova, Artemy D. Beniaminov, Maria S. Fedorova, Anastasiya V. Snezhkina, Anna V. Kudryavtseva, Alexey A. Dmitriev, Nataliya V. Melnikova

**Affiliations:** ^1^Engelhardt Institute of Molecular Biology, Russian Academy of Sciences, Moscow, Russia; ^2^Moscow Institute of Physics and Technology, Moscow, Russia

**Keywords:** *Populus*, poplar, sex, transcriptome sequencing, gene expression, DNA polymorphism, *CLC* gene

## Abstract

Transcriptome sequencing of leaves, catkin axes, and flowers from male and female trees of *Populus* × *sibirica* and genome sequencing of the same plants were performed for the first time. The availability of both genome and transcriptome sequencing data enabled the identification of allele-specific expression. Such an analysis was performed for genes from the sex-determining region (SDR). *P.* × *sibirica* is an intersectional hybrid between species from sections *Aigeiros* (*Populus nigra*) and *Tacamahaca* (*Populus laurifolia*, *Populus suaveolens*, or *Populus* × *moskoviensis*); therefore, a significant number of heterozygous polymorphisms were identified in the SDR that allowed us to distinguish between alleles. In the SDR, both allelic variants of the *TCP* (T-complex protein 1 subunit gamma), *CLC* (Chloride channel protein CLC-c), and *MET1* (DNA-methyltransferase 1) genes were expressed in females, while in males, two allelic variants were expressed for *TCP* and *MET1* but only one allelic variant prevailed for *CLC*. Targeted sequencing of *TCP*, *CLC*, and *MET1* regions on a representative set of trees confirmed the sex-associated allele-specific expression of the *CLC* gene in generative and vegetative tissues of *P.* × *sibirica*. Our study brings new knowledge on sex-associated differences in *Populus* species.

## Introduction

In contrast to animals, hermaphroditism is common in angiosperm plants—about 90% of these species produce bisexual flowers, about 5% are monoecious and have both male and female flowers on the same individual, and only about 5% are dioecious and have male and female flowers on separate individuals (Yampolsky and Yampolsky, 1922; Renner, 2014). Besides, there are gynodioecious plant species (with female and hermaphroditic individuals) and androdioecious species (with male and hermaphroditic individuals) that are less common (Bawa, 1980). It is known that dioecy can emerge from hermaphroditism directly or form *via* gynodioecy, androdioecy, monoecy, and, in some cases, heterostyly (Bawa, 1980). Evolution to dioecy involves a positive selection of mutations that lead to male and female sterility (Spigler and Ashman, 2012). In plants, divergence of sexes took place relatively recently and occurred fast, independently, and repeatedly, which led to very polymorphic mechanisms of sex determination (Charlesworth, 2002; Tanurdzic and Banks, 2004; Diggle et al., 2011; Bachtrog et al., 2014; Kafer et al., 2017). The genus *Populus* is presented by dioecious species and, due to extensive genetic studies (Tuskan et al., 2006; Jansson and Douglas, 2007; Jansson et al., 2010), is a promising object for research of differences between sexes. Poplars are wind-pollinated trees, and different species are easily crossed, resulting in the emergence of natural interspecific hybrids and a high level of genetic diversity (Rae et al., 2007; Roe et al., 2014; Jiang et al., 2016).

Most *Populus* species have an XY system of sex determination, while *Populus alba* has a ZW system. Sex-specific DNA polymorphisms were identified in *Populus* species, and the sex-determining region (SDR) was mapped to the pericentromeric region of chromosome 19 in aspens and the peritelomeric region of chromosome 19 in most studied poplars, except for *Populus euphratica* (the SDR is located on chromosome 14) (Gaudet et al., 2008; Yin et al., 2008; Pakull et al., 2009, 2011, 2015; Paolucci et al., 2010; Kersten et al., 2014; Geraldes et al., 2015; McKown et al., 2017; Melnikova et al., 2019; Müller et al., 2020; Xue et al., 2020; Yang et al., 2020; Zhou et al., 2020).

It was shown that *ARABIDOPSIS RESPONSE REGULATOR 17* (*ARR17*) ortholog plays a key role in sex determination. Involved in the cytokinin pathway, this gene is presented in genomes of males and females of *Populus* species with the XY system of sex determination, but its partial repeats were identified only in SDRs of the males. It was suggested that the locus of these repeats produces small RNAs that silence *ARR17 via* DNA methylation. *ARR17* works as a sex switch: male flowers are formed when *ARR17* is off and female ones when *ARR17* is on (Müller et al., 2020).

In the SDR of male *Populus deltoides*, two long Y-specific hemizygous sequences (YHSs) were identified: YHS1 was about 35 kb, and YHS2 was about 4.3 kb. YHS1 included two male-specific genes: one, named *FERR-R*, contained partial duplications of the *FERR* gene (named *ARR17* in the study of Müller et al., 2020) and repressed the formation of female generative organs, likely through the methylation of the *FERR* gene and cleavage of its transcript, and the other one, named *MSL*, belonged to the LTR/Gypsy transposon family and transcribed long non-coding RNA, which probably promoted the development of male reproductive organs of *P. deltoides*. Complete *MSL* sequence was also identified in males of *Populus simonii* but not *Populus davidiana* and *Populus tremula*. The SDR also contained genes encoding T-complex protein 1 subunit gamma (TCP), Chloride channel protein CLC-c (CLC), and DNA-methyltransferase 1 (MET1), which were present in both males and females of *P. deltoides* (Xue et al., 2020).

In *Populus trichocarpa*, about 50 kb of its SDR were identified as male-specific (these sequences were absent in female plants). The SDR also contained five genes, which were present in both males and females of *P. trichocarpa*—genes encoding TCP, CLC, MET1, and leucine-rich repeat-containing protein (NB-ARC), and also an unknown gene. Genomic sites that were homozygous in females and heterozygous in males were revealed, and alternative Y and X haplotypes were identified (Zhou et al., 2020).

Differences in gene expression between male and female poplars and aspens were also studied. Overexpression of a gene that is necessary for the formation of female reproductive organs (named *ARR17*, *FERR*, or *RR* in different studies) was revealed in female genotypes at early stages of flower development (Cronk et al., 2020; Müller et al., 2020; Xue et al., 2020; Yang et al., 2020), while the expression of *MSL* was male-specific and continuous (Xue et al., 2020). Sex-specific gene expression was observed in flowers but not in leaves of *Populus balsamifera*: female-biased genes were related to photosynthesis, while male-biased genes were related to mitochondria (Sanderson et al., 2019). The analysis of transcriptomic data for male and female reproductive organs of *P. balsamifera* revealed gene expression trajectories during flower development and male-biased expression of two MADS-box genes, *APETALA3* and *PISTILLATA* (Cronk et al., 2020). Besides, in several studies of *Populus* species, sex-associated differences in gene expression were observed under particular, predominantly unfavorable, conditions (Melnikova et al., 2017; Han et al., 2018; Song et al., 2019).

Despite the significant improvement in our understanding of sex determination in *Populus* species, our knowledge of sex-specific differences in this genus is still incomplete. Further studies concerning male and female distinctions in poplars and aspens are necessary, and genomic and transcriptomic data for different species of the genus *Populus* should be obtained and analyzed. In the present study, to identify sex differences, we performed genome and transcriptome sequencing for male and female plants of one of the most common poplars in the cities of central Russia—*Populus* × *sibirica*, which is an intersectional hybrid likely between species from section *Aigeiros* (*Populus nigra*) and section *Tacamahaca* (the exact progenitor is still unknown, it could be *Populus laurifolia*, *Populus suaveolens*, or *Populus* × *moskoviensis*) (Mayorov et al., 2012; Kostina and Nasimovich, 2014; Kostina et al., 2017).

## Materials and Methods

### Plant Material

We used plant material of *P.* × *sibirica* trees growing in Moscow within the territory from 55°41′29″N to 55°42′35″N and from 37°33′33″E to 37°35′26″E. Leaves, catkin axes, and flowers were collected from male and female plants during the beginning of flowering, immediately frozen in liquid nitrogen, and stored at −70°C until further use. Samples from one male and one female trees were used for whole-genome and transcriptome sequencing, and samples from 10 male and 10 female trees for targeted sequencing of DNA and cDNA.

### RNA Extraction and Transcriptome Sequencing

For RNA extraction, the Quick-RNA Miniprep Kit (Zymo Research, Irvine, CA, United States) was used. Plant samples (three for each tissue) were homogenized in a lysis buffer with solid glass beads (Sigma-Aldrich, St. Louis, MO, United States) using MagNA Lyser (Roche, Basel, Switzerland), and further manipulations were performed following the manufacturer’s protocol with DNase I treatment step. RNA quality and concentration were evaluated on 2100 Bioanalyzer (Agilent Technologies, Santa Clara, CA, United States) with Agilent RNA 6000 Nano Kit (Agilent Technologies) and Qubit 2.0 (Life Technologies, Carlsbad, CA, United States) with Qubit RNA BR Assay Kit (Life Technologies) respectively.

For cDNA library preparation from 1 μg of total RNA, the NEBNext Poly(A) mRNA Magnetic Isolation Module (New England Biolabs, Hitchin, United Kingdom) and NEBNext Ultra II Directional RNA Library Prep Kit for Illumina (New England Biolabs) were used. cDNA library quality and concentration were evaluated on 2100 Bioanalyzer (Agilent Technologies) with Agilent DNA 1000 Kit (Agilent Technologies) and Qubit 2.0 (Life Technologies) with Qubit dsDNA HS Assay Kit (Life Technologies) respectively. Transcriptome sequencing of leaves, catkin axes, and flowers of male and female *P.* × *sibirica* plants (in total, six libraries) was performed on NextSeq 500 (Illumina, San Diego, CA, United States) with a read length of 86 bp.

### DNA Extraction and Whole-Genome Sequencing

*Populus* × *sibirica* leaves were homogenized in the lysis buffer from the DNeasy Plant Mini Kit (Qiagen, Germantown, MD, United States) with solid glass beads (Sigma-Aldrich) using MagNA Lyser (Roche), then, DNA extraction was performed following the manufacturer’s protocol. The extracted DNA was fragmented on an S220 ultrasonic homogenizer (Covaris, Woburn, MA, United States), and 1 μg of fragmented DNA was used to prepare the library using the NEBNext Ultra II DNA Library Prep Kit for Illumina (New England Biolabs) according to the manufacturer’s protocol with size selection of adapter-ligated DNA of about 600 bp. The quality and concentration of DNA libraries were evaluated using 2100 Bioanalyzer (Agilent Technologies) with Agilent DNA 1000 Kit (Agilent Technologies) and Qubit 2.0 (Life Technologies) with Qubit dsDNA HS Assay Kit (Life Technologies) respectively. DNA libraries were sequenced on HiSeq 2500 (Illumina) with a read length of 125 + 125 bp.

### Targeted Genome and Transcriptome Sequencing

DNA was extracted from leaves, and RNA was extracted from leaves, catkin axes, and flowers of 10 male and 10 female plants of *P.* × *sibirica* as described above. One microgram of RNA for each sample was treated with DNase I (Thermo Fisher Scientific, Waltham, MA, United States) and used for reverse transcription with random hexamer primers (Evrogen, Moscow, Russia) and Mint reverse transcriptase (Evrogen) following the manufacturer’s protocol. Primers were designed for amplification of parts of the *TCP*, *CLC*, and *MET1* genes and further sequencing on the Illumina platform as described in our previous works (Melnikova et al., 2019; Dmitriev et al., 2020; Novakovskiy et al., 2020). In brief, two PCRs were used for the library preparation. The first PCR enabled amplification of target gene regions and the addition of universal adapters to amplicons according to the Illumina protocol^1^. In the second PCR, Nextera XT v2 index primers containing dual-index barcodes and sequencing adapters were used. Primer sequences are listed in Supplementary Data 1. For each of the 20 genotypes, the amplicons were obtained independently for DNA from leaves and cDNA from leaves, catkin axes, and flowers at the first PCR and then labeled with unique sample-specific indexes at the second PCR.

### Data Analysis

For the expression analysis, RNA-Seq reads were trimmed using Trimmomatic 0.32 (Bolger et al., 2014), mapped to the male *P. trichocarpa* ‘‘Stettler 14’’ genome^2^ (Hofmeister et al., 2020) using STAR 2.8 (Dobin et al., 2013), and then quantified (a) for the annotated genes using the featureCounts tool from the Subread package 1.6.0 (Liao et al., 2014) and (b) for the region Chr18:16,200,000–16,320,000 (200-bp intervals) corresponding to the SDR of the male *P. trichocarpa* “Stettler 14” (Zhou et al., 2020) with BEDTools 2.26.0 (Quinlan and Hall, 2010). It should be noted that in the “Stettler 14” genome assembly, the SDR is located on chromosome 18, but the genetic map showed that its correct place is on chromosome 19 (Zhou et al., 2020). The derived read count values were analyzed using edgeR 3.28.1 (for R 3.6.3) (Robinson et al., 2010). TMM normalization and quasi-likelihood *F*-test were applied. Pairwise distance (dissimilarity) was calculated as “1 – *r*,” where *r* is the Spearman’s rank correlation coefficient between gene expression profiles. For distance calculation, we used genes with an average CPM (counts per million) >16.

For the identification of polymorphisms in the SDR of *P.* × *sibirica* based on the obtained sequencing data, reads were trimmed using Trimmomatic and mapped to the male *P. trichocarpa* “Stettler 14” genome with BWA-MEM 0.7.17 (Li, 2013). The derived BAM files were preprocessed with Picard tools 2.21.3, including verification of mate information (the FixMateInformation tool), marking duplicated reads [the MarkDuplicatesWithMateCigar tool; only for whole-genome sequencing (WGS) paired-end reads], or splitting reads spanning introns (SplitNCigarReads from GATK 4.1.9.0; only for RNA-Seq reads). Variant calling was performed using FreeBayes 1.3.2 (Garrison and Marth, 2012) for the SDR of the male *P. trichocarpa* “Stettler 14.” We filtered out variant candidates with Phred quality <15.

## Results

Transcriptome sequencing of leaves, catkin axes, and flowers of male and female *P.* × *sibirica* plants produced from 10.7 to 12.8 million 86-bp single-end reads for each sample. WGS gave 36 million paired-end 2 × 125-bp reads for the male poplar and 52 million reads for the female one that corresponded to about 18× and 26× genome coverage respectively. The raw data were deposited in the NCBI Sequence Read Archive (SRA) under the BioProject accession number PRJNA644206.

**FIGURE 1:**
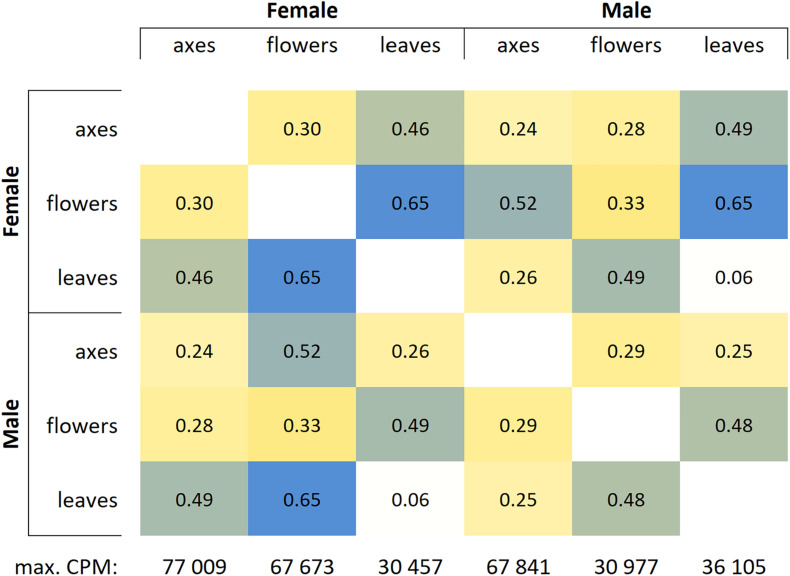
Distance matrix for gene expression profiles in leaves, catkin axes, and flowers of male and female *Populus* × *sibirica* plants. Transcriptome sequencing data; genes with CPM (counts per million) >16 were taken into account; max. CPM, maximum CPM among all genes.

**FIGURE 2:**
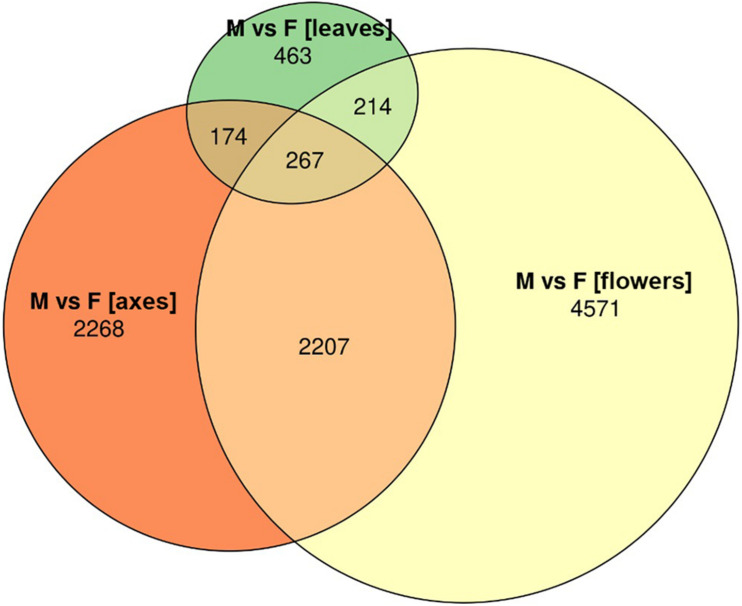
Euler diagram for genes differentially expressed between male and female *Populus* × *sibirica* plants in leaves, catkin axes, and flowers. Transcriptome sequencing data; genes with CPM (counts per million) >8 and at least twofold expression level differences were taken into account; M, male; F, female.

Based on RNA-Seq data, we assessed distinctions in gene expression profiles between different male and female *P.* × *sibirica* tissues. Multidimensional scaling plots (Supplementary Data 2, 3) and a distance matrix (Figure 1) showed that the expression profiles of male and female leaves differed much less than those of flowers and catkin axes. So, when comparing male and female leaves, we revealed 1,118 differentially expressed genes (DEGs), having at least twofold expression level differences and a sufficient expression level (CPM > 8), while for catkin axes and flowers, 4,916 and 7,259 DEGs were identified (Figure 2). Thus, sex did not result in such significant differences in the transcriptomes of vegetative organs, as it happened for generative ones. These results are in concordance with those from previous studies in *P. balsamifera* that revealed more significant expression differences between male and female flowers compared to the leaves (Sanderson et al., 2019).

Based on whole-genome and transcriptome sequencing data, coverage profiles and polymorphisms were evaluated for the SDR of *P.* × *sibirica* using *P. trichocarpa* “Stettler 14” genome assembly as a reference [as mentioned above, the SDR in this assembly is located on chromosome 18, but its correct place is on chromosome 19 (Zhou et al., 2020); however, the SDR sequence itself is complete that allows us to use it for the analysis]. The results of the analysis are presented in Supplementary Data 4. As in previous studies on SDRs of male poplars (Müller et al., 2020; Xue et al., 2020; Zhou et al., 2020), we revealed male-specific genomic regions, the coverage of which was identified only within genomic data of the male; however, expression from them was not observed (Supplementary Data 4). This can be associated with the type of plant material used in the present study (particular tissue and stage of development).

A more detailed analysis was performed for five genes, which were identified in the SDR of *P. trichocarpa* “Stettler 14” in the study of Zhou et al. (2020): *TCP* (PtStettler14.18G127900, Chr18:16,275,593-16,279,859, reverse), *CLC* (PtStettler14.18G127800, Chr18:16,268,169-16,272,894, reverse), *MET1* (PtStettler14.18G127700, Chr18:16,249,745-16,259,190, reverse), *NB-ARC* (PtStettler14.18G127600, Chr18:16,226,313-16,236,215, reverse), and an unknown gene (PtStettler14.18G127500, Chr18:16,214,199-16,215,600, forward) (Zhou et al., 2020). Three of these genes (*TCP*, *CLC*, and *MET1*) were expressed in *P.* × *sibirica* tissues under study (leaves, catkin axes, and flowers) that enabled the identification of polymorphisms in their transcripts and further comparison with polymorphisms from our genome sequencing data. *P.* × *sibirica* is an intersectional hybrid (Mayorov et al., 2012; Kostina and Nasimovich, 2014; Kostina et al., 2017), so a significant number of heterozygous polymorphisms were identified in the SDR. In the tissues of the female, two variants of nucleotides were revealed in transcriptomic data in sites where two variants of nucleotides were found in genomic data; thus, both allelic variants of *TCP*, *CLC*, and *MET1* genes were expressed in the female (Supplementary Data 5). In the male, we observed the same for *TCP* and *MET1* but not *CLC*. For this gene, only one nucleotide variant prevailed in male transcriptomic data for leaves, catkin axes, and flowers in sites that had two variants of nucleotides in male genomic data (Supplementary Data 6).

**FIGURE 3:**
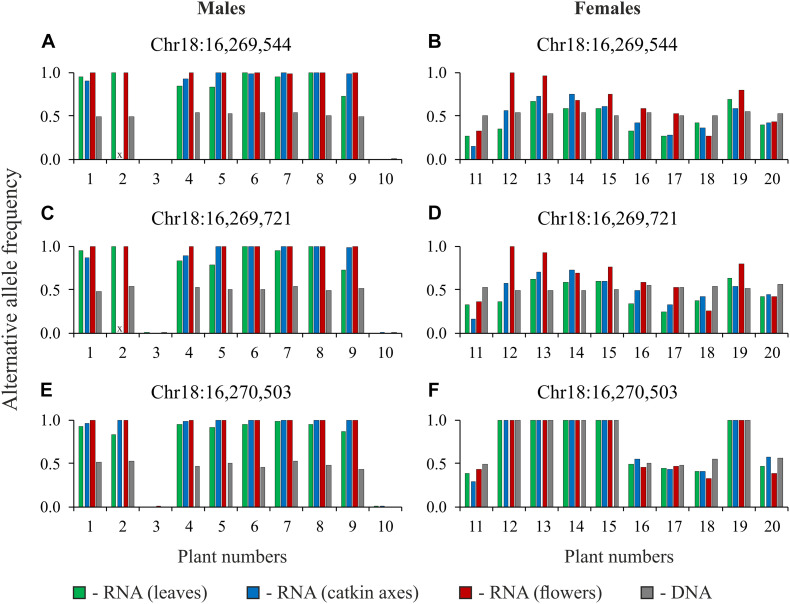
Alternative allele frequencies for heterozygous sites of the *CLC* gene for DNA and cDNA (RNA from leaves, catkin axes, and flowers) samples of 10 male **(A,C,E)** and 10 female **(B,D,F)** plants of *Populus* × *sibirica*. Targeted sequencing data; chromosome number and nucleotide position are given above each pair of charts according to the *P. trichocarpa* “Stettler 14” genome assembly; plant numbers (1–20) correspond to plant numbers in Supplementary Data 7; x, no data.

To confirm our results, we performed targeted sequencing of *TCP*, *CLC*, and *MET1* gene regions for DNA and cDNA (RNA extracted from leaves, catkin axes, and flowers) samples of 10 male and 10 female plants of *P.* × *sibirica* (in total, 80 samples). It should be noted that two primer pairs for the *CLC* gene amplified regions with introns. Such an approach enabled us to control the absence of DNA in RNA samples through the analysis of sequencing data. The results of variant calling in amplicons of the *TCP*, *CLC*, and *MET1* genes are presented in Supplementary Data 7. When two variants of nucleotides (frequencies of alternative and reference alleles about 0.5) were revealed in a particular site of the *TCP* or *MET1* amplicons obtained from DNA, two variants of nucleotides (with significant frequencies of both alternative and reference alleles) were also revealed in the amplicons obtained from cDNA (RNA from leaves, catkin axes, and flowers) of the same genotype for most males and females. As expected, the same was observed for the *CLC* gene in females. However, for *CLC* in males, in sites with frequencies of alternative and reference alleles about 0.5 for the amplicons from DNA, only one nucleotide variant dominated (frequency of an alternative allele or a reference one was close to 1) in the amplicons from cDNA (RNA from leaves, catkin axes, and flowers) of the same genotype. The sets of heterozygous sites in the *TCP*, *CLC*, and *MET1* genes were different between males and females of *P.* × *sibirica* that is probably associated with suppression of recombination in the SDR. However, some common heterozygous sites were also revealed, and data for three of such sites of the *CLC* gene are presented in Figure 3 as an illustration of the sex-associated allele-specific expression of this gene.

## Discussion

Due to recent studies on the genetics of sex in *Populus* species, especially obtaining high-quality genome assemblies of male individuals (Geraldes et al., 2015; McKown et al., 2017; Xue et al., 2020; Yang et al., 2020; Zhou et al., 2020), a detailed investigation of SDRs became possible. In the present work, the analysis of the SDR of *P.* × *sibirica* was performed for the identification of sex-specific differences. Based on the comparison of whole-genome and transcriptome sequencing data for male and female plants of *P.* × *sibirica*, we evaluated allele frequencies in heterozygous sites of genes from the SDR. Since *P.* × *sibirica* is an intersectional hybrid, a significant number of heterozygous sites were revealed that enabled us to identify the predominant expression of the *CLC* gene only from one allele in generative and vegetative tissues of the male but not female. Then, we used targeted sequencing of DNA and cDNA samples of 20 *P.* × *sibirica* plants (10 males and 10 females) and confirmed the sex-associated allele-specific expression of the *CLC* gene in this poplar. Moreover, based on obtained by us high-quality genome assembly of male *P.* × *sibirica* with separated Y and X haplotypes of the SDR (unpublished data), it can be concluded that it is the Y SDR haplotype with the suppressed *CLC* expression.

The expression of genes from the SDR, including *CLC*, was analyzed in previous studies of poplars. In *P. balsamifera*, *CLC* upregulation was revealed in flowers of females compared to those of males (Sanderson et al., 2019). Opposite expression polarity was revealed during the development of reproductive organs in male and female *P. balsamifera* for two variants of *TCP*, *CLC*, and *MET1* (located on both chromosome 18 and scaffold 42) when the genome of female *P. trichocarpa* was used as a reference (Cronk et al., 2020). However, no consistent differences in the expression of the *TCP*, *CLC*, and *MET1* genes were revealed between male and female *P. deltoides* in developing flowers (Xue et al., 2020). Thus, for the first time, we revealed sex-associated allele-specific expression for one of the three genes from the SDR (for *CLC* but not for *TCP* and *MET1*) in generative and also vegetative tissues of poplar, namely *P.* × *sibirica*.

*CLC* genes participate in Cl^–^ and NO_3_^–^ transport, are involved in response to salt stress, and are related to the efficiency of nitrogen use in plants (Liao et al., 2018; Liu et al., 2020; Subba et al., 2020). Therefore, differences in *CLC* expression in vegetative tissues of *P.* × *sibirica* could be of interest in terms of determination of the possible role of sex in adaptation to particular environments. A significant number of studies concerning differences in stress response of male and female plants of *Populus* species were performed; however, there is no consensus on the association of stress resistance with sex (Melnikova et al., 2017). The increased transcription level of *CLC* was revealed under high salinity conditions in female poplars but not in male ones, and the authors suggested that males had more efficiency in Cl^–^ homeostasis than females (Jiang et al., 2012). Besides, under salt stress, *CLC* was upregulated in *Populus pruinosa*, which is distributed in deserts with underground water close to the surface, but not in *P. euphratica*, which occurs in deserts with deep underground water (Zhang et al., 2014). It was also shown that overexpression of the *CLC* gene from soybean in transgenic poplars improved salt tolerance (Sun et al., 2013).

Thus, the *CLC* gene is involved in salt stress response in poplars, and our data on its sex-associated allele-specific expression in vegetative tissues of *P.* × *sibirica* suggest that differences between male and female poplars could take place not only in generative organs but also in whole plants and are implicated in stress resistance. However, further research is needed to clarify this issue.

## Conclusion

Comprehensive genetic and epigenetic studies of phylogenetically distant *Populus* species are essential for the identification of male- and female-specific differences and sex-associated pathways. In our study, transcriptome sequencing of leaves, catkin axes, and flowers from male and female trees of *P.* × *sibirica* and genome sequencing of the same plants were performed for the first time. In the SDR, both allelic variants of the *TCP*, *CLC*, and *MET1* genes were expressed in females, while, in males, both allelic variants were expressed for *TCP* and *MET1*, but only one variant prevailed for the *CLC* gene. Targeted sequencing of *TCP*, *CLC*, and *MET1* gene regions obtained from DNA and cDNA (RNA extracted from leaves, catkin axes, and flowers) samples of 10 male and 10 female plants of *P.* × *sibirica* confirmed the predominant expression of only one allelic variant of the *CLC* gene in males. Generalization of the currently available data on *CLC* expression allows us to suggest that the identified sex-associated allele-specific expression of this gene in both generative and vegetative organs can be involved in different salt resistance of males and females of *Populus* species.

## Data Availability Statement

The obtained sequencing data can be found in NCBI under the BioProject accession number PRJNA644206.

## Author Contributions

AD and NM conceived and designed the work. EP, RN, LP, AB, MF, and AS performed the experiments. EP, GK, VL, ED, AK, AD, and NM analyzed the data. EP, GK, ED, AD, and NM wrote the manuscript. All authors read and approved the final manuscript.

## Conflict of Interest

The authors declare that the research was conducted in the absence of any commercial or financial relationships that could be construed as a potential conflict of interest.

## Publisher’s Note

All claims expressed in this article are solely those of the authors and do not necessarily represent those of their affiliated organizations, or those of the publisher, the editors and the reviewers. Any product that may be evaluated in this article, or claim that may be made by its manufacturer, is not guaranteed or endorsed by the publisher.

## Footnotes

1
https://support.illumina.com/documents/documentation/chemistry_documentation/16s/16s-metagenomic-library-prep-guide-15044223-b.pdf

2
https://phytozome-next.jgi.doe.gov/info/PtrichocarpaStettler14_v1_1

## Supplementary Material

Supplementary Data 1 Primers for the first stage of DNA library preparation.

Supplementary Data 2 Multidimensional scaling plot (dimensions 1 and 2) for gene expression profiles in leaves, catkin axes, and flowers of male and female *Populus* × *sibirica* plants.

Supplementary Data 3 Multidimensional scaling plot (dimensions 1 and 3) for gene expression profiles in leaves, catkin axes, and flowers of male and female *Populus* × *sibirica* plants.

Supplementary Data 4 Polymorphisms in the SDR (including *TCP*, *CLC*, and *MET1* genes) for male and female *Populus* × *sibirica* plants based on genome and transcriptome sequencing data.

Supplementary Data 5 The selected polymorphisms in the *TCP*, *CLC*, and *MET1* genes for the female *Populus* × *sibirica* plant based on genome and transcriptome sequencing data.

Supplementary Data 6 The selected polymorphisms in the *TCP*, *CLC*, and *MET1* genes for the male *Populus* × *sibirica* plant based on genome and transcriptome sequencing data.

Supplementary Data 7 Polymorphisms in the *TCP*, *CLC*, and *MET1* genes for male and female *Populus* × *sibirica* plants based on targeted sequencing data.
