# Supplementary material for: Genome and Transcriptome Sequencing of Populus × sibirica Identified Sex-Associated Allele-Specific Expression of the CLC Gene
Source: Front Genet. 2021 Aug 11;12:676935. doi: 10.3389/fgene.2021.676935 (PMC8385651; doi:10.3389/fgene.2021.676935)
Supplement: Supplementary Data 1 — Primers for the first stage of DNA library preparation. [file Data_Sheet_1.DOCX]

**Supplementary Data 1. Primers for the first stage of DNA library preparation.** Overhang Illumina adapter sequences are marked with blue and orange, sequences that are necessary for amplification of target regions are marked with green. i7 – Nextera Illumina 8-base Index 1, i5 – Nextera Illumina 8-base Index 2.

| Primer pair | Sequence |
| --- | --- |
| MET1_2903-3355_F  MET1_2903-3355_R | TCGTCGGCAGCGTCAGATGTGTATAAGAGACAGCAAGGAAGAGCCTACTGGTGG  GTCTCGTGGGCTCGGAGATGTGTATAAGAGACAGCCAGCAGGCTTAATGCAGTC |
| CLC_4342-4693_F  CLC_4342-4693_R | TCGTCGGCAGCGTCAGATGTGTATAAGAGACAGCAGAGACCTCCAATTGTTGGGA  GTCTCGTGGGCTCGGAGATGTGTATAAGAGACAGCCCGATTATGAGATGGTTTCACA |
| CLC_4128-4694_F  CLC_4128-4694_R | TCGTCGGCAGCGTCAGATGTGTATAAGAGACAGTCGACAAATTGGTCTCAGGCA  GTCTCGTGGGCTCGGAGATGTGTATAAGAGACAGTCCCGATTATGAGATGGTTTCACA |
| CLC_3106-3595_F  CLC_3106-3595_R | TCGTCGGCAGCGTCAGATGTGTATAAGAGACAGTGTTCTTACGGCCTGCCTTG  GTCTCGTGGGCTCGGAGATGTGTATAAGAGACAGACATCACAAGGGGAAGCAACA |
| CLC_2334-2882_F  CLC_2334-2882_R | TCGTCGGCAGCGTCAGATGTGTATAAGAGACAGCTGGTGCATGCATAGCCTCT  GTCTCGTGGGCTCGGAGATGTGTATAAGAGACAGAGAGTATCACAGCTAAAACATCCG |
| TCP_3681-3961_F  TCP_3681-3961_R | TCGTCGGCAGCGTCAGATGTGTATAAGAGACAGTGCCTACAATGTCAAGGCACA  GTCTCGTGGGCTCGGAGATGTGTATAAGAGACAGTCAAAACTTGGGACAAAACAGC |
| TCP_1792-2342_F  TCP_1792-2342_R | TCGTCGGCAGCGTCAGATGTGTATAAGAGACAGACCTCAATAGTTGGTGTGGACC  GTCTCGTGGGCTCGGAGATGTGTATAAGAGACAGGCAAAGAATTCATCCCCAATTTTCT |
| Nextera XT v2 (i5) Nextera XT v2 (i7) | AATGATACGGCGACCACCGAGATCTACAC[i5]TCGTCGGCAGCGTC CAAGCAGAAGACGGCATACGAGAT[i7]GTCTCGTGGGCTCGG |
