# Supplementary material for: Genome and Transcriptome Sequencing of Populus × sibirica Identified Sex-Associated Allele-Specific Expression of the CLC Gene
Source: Front Genet. 2021 Aug 11;12:676935. doi: 10.3389/fgene.2021.676935 (PMC8385651; doi:10.3389/fgene.2021.676935)
Supplement: Supplementary Data 2 — Multidimensional scaling plot (dimensions 1 and 2) for gene expression profiles in leaves, catkin axes, and flowers of male and female Populus × sibirica plants. [file Data_Sheet_2.PDF]

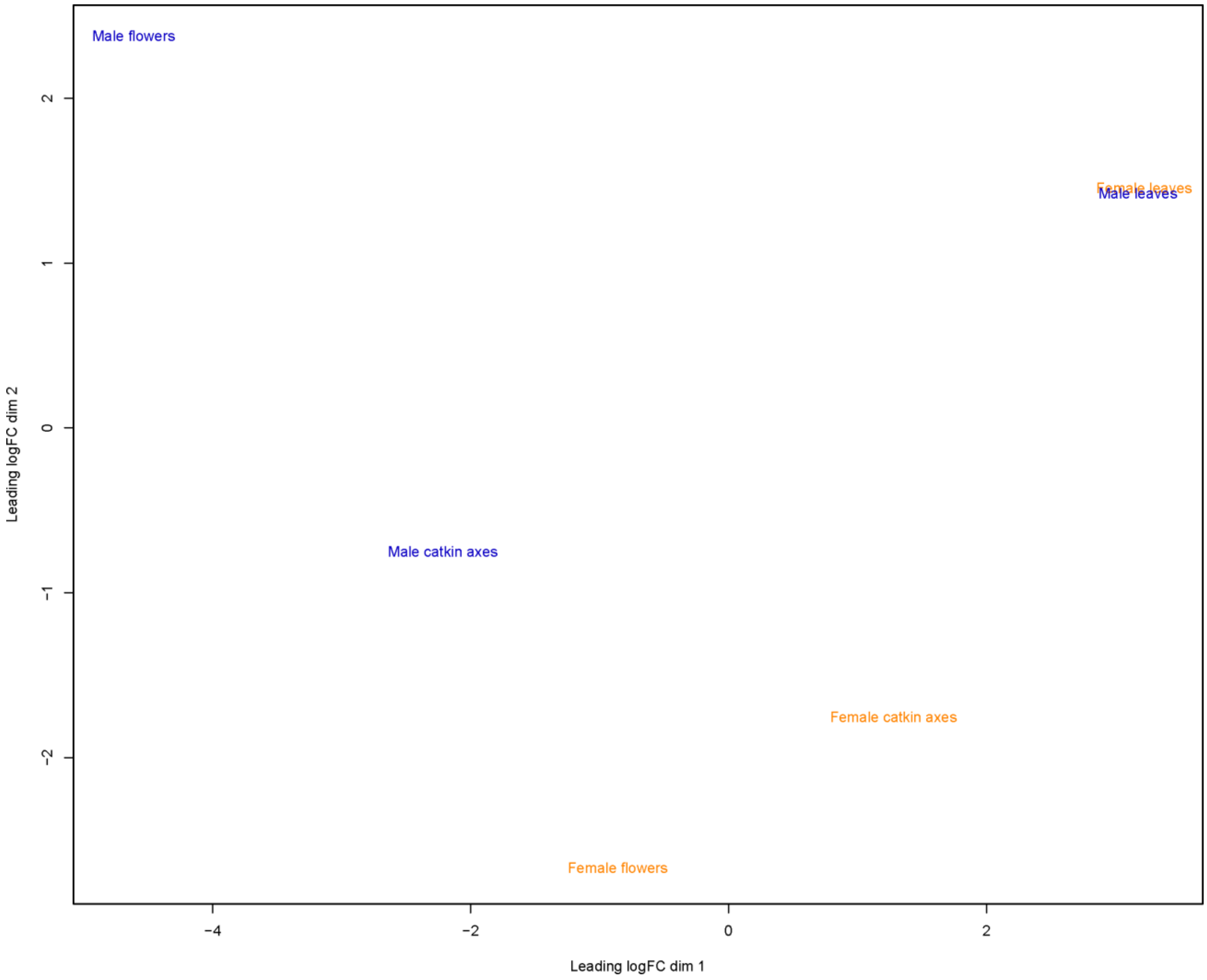

Supplementary Data 2. Multidimensional scaling plot (dimensions 1 and 2) for gene expression profiles in leaves, catkin axes, and flowers of male and female *Populus* x *sibirica* plants. Male samples – blue, female samples – orange.
